# Birds of a feather flock together: structural characterization of red-crowned crane and turkey aveparvoviruses

**DOI:** 10.1128/jvi.00110-25

**Published:** 2025-07-03

**Authors:** Jane Hsi, Mario Mietzsch, Matthew Patney, Sunny Chen, Anjelique Sawh-Gopal, Paul Chipman, Robert McKenna

**Affiliations:** 1 Department of Biochemistry and Molecular Biology, Center for Structural Biology, McKnight Brain Institute, College of Medicine, University of Florida 12233, Gainesville, Florida, USA; 2 The Herbert Wertheim UF Scripps Institute for Biomedical Technology & Innovation 3463, Jupiter, Florida, USA; Cornell University Baker Institute for Animal Health, Ithaca, New York, USA

**Keywords:** parvovirus, *Aveparvovirus*, capsid, cryo-EM, pathogen, RCPV, TuPV, red-crowned crane, turkey

## Abstract

The parvoviruses have non-enveloped *T* = 1 icosahedral capsids that are ~25 nm in diameter, which package linear single-stranded DNA and infect a wide range of hosts. To date, parvoviruses affecting birds have been identified in two genera: *Ave*- and *Dependoparvovirus*, and no capsid structures have been determined for any member of *Aveparvovirus*. This study investigates two bird viruses of this genus: red-crowned crane parvovirus (RCPV) and turkey parvovirus (TuPV). While the pathogenicity of RCPV is currently unknown, TuPV has been associated with gastrointestinal diseases, especially in juvenile birds. High-resolution structures of the RCPV and TuPV capsids were determined by cryo-electron microscopy at a resolution of 2.66 Å and 2.35 Å, respectively. A structural comparison of the RCPV and TuPV capsids shows that they exhibit many conserved features, such as a channel at the five-fold symmetry axis and surface depressions at the two-fold axis, as previously observed in parvoviruses from other genera. However, major structural differences were observed at the three-fold axes, with both RCPV and TuPV displaying recessed protrusions. In addition, terminal sialic acid was identified as a potential glycan receptor for RCPV. This study extends the architectural portfolio of structural parvovirology and will provide insights into parvoviral diversity and characterization of these viruses.

**IMPORTANCE:**

This study presents the capsid structures of two aveparvoviruses, red-crowned crane parvovirus (RCPV) and turkey parvovirus (TuPV), extending the structural repertoire of the *Parvoviridae*. While the pathogenicity of RCPV is unknown, the red-crowned cranes are among the rarest crane species. To date, very few virological studies have been conducted for this rare avian species, and understanding their virome could contribute to conservation efforts. Additionally, several studies have previously suggested that TuPV is associated with cases of enteric disease syndrome. To date, no commercial antivirals or vaccines are available for TuPV. The structural characterization of its capsid may contribute toward the development of a treatment to control the spread of infection.

## INTRODUCTION

Birds can be reservoirs for zoonotic viruses that may negatively impact human health and agriculture, and avian influenza A virus is one of several examples (1). These bird viruses co-circulate within avian populations, which may result in the emergence of new viruses through potential reassortment or recombination.

Parvoviruses are linear single-stranded DNA (ssDNA) viruses with *T* = 1 icosahedral symmetry capsids, affecting both vertebrate and invertebrate hosts (2). *Parvoviridae* is divided into three subfamilies: *Parvovirinae*, containing genera that infect vertebrate hosts; *Densovirinae*, infecting invertebrate hosts; and *Hamaparvovirinae*, infecting both vertebrate and invertebrate hosts (3). Currently, the *Parvovirinae* subfamily contains 11 genera: *Amdo-*, *Arti-*, *Ave-*, *Boca-*, *Dependo-*, *Erythro-*, *Copi-*, *Lori-*, *Proto-*, *Sande-*, and *Tetraparvovirus* (3). Among these 11 genera of *Parvovirinae*, several species from the *Aveparvovirus* and *Dependoparvovirus* genera have been reported to infect avian hosts. Many of the avian parvoviruses have been identified as significant pathogens affecting various avian species worldwide in both wild and domesticated birds. Over the years, research on avian parvoviruses has identified diverse species with distinct genetic characteristics, clinical manifestations, and epidemiological patterns (4–6).

Dependoparvoviruses affecting waterfowl hosts, such as goose parvovirus (GPV) and Muscovy duck parvovirus (MDPV), are capable of autonomous replication in the tissues of young goslings and ducklings. Both GPV and MDPV are considered the causative agents of Derzsy’s disease, with morbidity and mortality rates ranging from 70% to 100% in waterfowl that are between 3 and 4 weeks old (7–9). On the other hand, quail adeno-associated viruses (QAAV) are nonpathogenic dependoparvoviruses that require co-infection with a helper virus (adenovirus or herpesvirus) for effective infection in avian hosts (10).

Currently, there are six members in the *Aveparvovirus* genus, all of which have been detected in birds: chicken parvovirus (ChPV), turkey parvovirus (TuPV), red-crowned crane parvovirus (RCPV), pileated finch aveparvovirus (PfPV), pigeon parvovirus (PiPV), and macaw aveparvovirus (MAPV) (4, 11–13). In the 1980s, ChPV and TuPV were identified to infect juvenile chickens and turkeys, respectively, and as potential causative agents of enteric disorders, affecting growth rates in poultry worldwide (14, 15). Enteric disorders in poultry are frequently associated with the runting-stunting syndrome (RSS), poult enteritis mortality syndrome (PEMS), and poult enteritis complex (16). The disease mechanism for these intestinal pathogenic viruses is not well understood, and currently, there are no vaccines available to treat RSS and PEMS (17).

RCPV was first identified and characterized as part of a 2018 viral metagenomics study from the fecal virome of wild and domestic red-crowned crane populations in China (18). In the analysis, there were six nearly complete genomes belonging to *Parvoviridae*, including four new members of the genus *Chaphamaparvovirus* and two members of the genus *Aveparvovirus*. Red-crowned cranes are among the rarest crane species in the world, due to loss of habitat and infectious diseases. Very few virological studies have been conducted on red-crowned cranes, and insights from these studies could contribute to conservation efforts for these rare species (19).

Comparative genome analyses of aveparvoviruses reveal differences in genome length, ranging from ~4.6 to 5.5 kb of linear ssDNA genome, with flanking terminal repeats at the 5′ and 3′ ends, which are involved in replication and genome packaging (20). Between the terminal repeats are three open reading frames (ORFs): NS, NP, and VP. Aveparvoviruses have two promoters and a single polyadenylation site that regulates the transcription of non-structural and structural genes (21, 22). The NS ORF encodes for a protein with an endonuclease, a superfamily 3 domain helicase, rolling circle replication initiator protein motifs, and a DNA binding domain (23–27). These features are highly conserved among all parvoviruses and play a central role in DNA replication (11, 28, 29). A small middle ORF has been predicted to encode the NP protein, which is required for efficient viral DNA replication and to control capsid protein expression (30). The CAP ORF encodes the capsid proteins VP1 (viral protein) and VP2, with a size of ~77 kDa and ~60 kDa, respectively (15, 21, 31). VP1 and VP2 overlap at the C-terminus, with VP2 completely contained in VP1. At the N-terminus, VP1 possesses a unique region (VP1u) of ~140 amino acids. Sixty VPs assemble together to form an icosahedral capsid. Unlike most parvoviruses, aveparvoviruses lack an identified PLA_2_ domain in their VP1u (11).

Canine parvovirus (CPV), a member of the genus *Protoparvovirus*, was the first parvovirus capsid structure determined in 1991 (32). Since, more than 100 structures have been determined for members of the *Parvovirinae*, utilizing either x-ray crystallography or cryogenic-electron microscopy (cryo-EM) (31). Parvoviruses have *T* = 1 icosahedral capsids, assembled from 60 VP monomers. The conserved *Parvovirinae* VP core structure includes an eight-stranded (βB–βI) anti-parallel β-barrel motif, also called a jelly roll motif, in addition to a βA strand and an α helix. While these features are located on the interior of the capsid, the loops are inserted between the β-strands forming the exterior surface of the capsid. The apices of these loops display the most differences and are defined as variable regions (VRs), if two or more amino acids have Cα positions that differ greater than 2 Å when their VPs are superposed (33, 34). Despite the differences in their surface loops, all parvovirus capsids are assembled via two-, three-, and fivefold symmetry-related VP interactions and share the same characteristic features. At the fivefold symmetry axis, five loops form the cylindrical channels which are suggested to be the route for genome packaging/ejection and VP1 externalization (35, 36). The protrusions surrounding the threefold symmetry axis usually consist of multiple loops forming the morphology. Depressions are found at the twofold symmetry axis that is separated by raised regions known as two-/fivefold walls. Both variations in amino acid sequences and structure are responsible for determining receptor usage and host/tissue tropism (37, 38).

To date, capsid structures have been determined for viruses of 6 of the 11 genera of *Parvovirinae*. Currently, no capsid structures are known for five genera, including *Aveparvovirus*. In this study, the first *Aveparvovirus* structures, RCPV and TuPV, were determined utilizing cryo-EM and 3D image reconstruction to 2.66 Å and 2.35 Å resolution, respectively. Although comparisons of the *Aveparvovirus* capsids to members of the other *Parvovirinae* genera exhibit significant differences in the surface loops, the capsid core remains conserved among all parvoviruses. Furthermore, RCPV was found to bind sialic acid, which is a common glycan receptor utilized by many other parvoviruses. The structural and preliminary functional characterization presented here will expand upon the structural portfolio of parvoviruses in addition to aiding in the design of vaccines or antivirals for the avian species negatively impacted by these viruses.

## RESULTS AND DISCUSSION

### RCPV and TuPV capsids can be expressed in Sf9 cells

**Fig 1:**
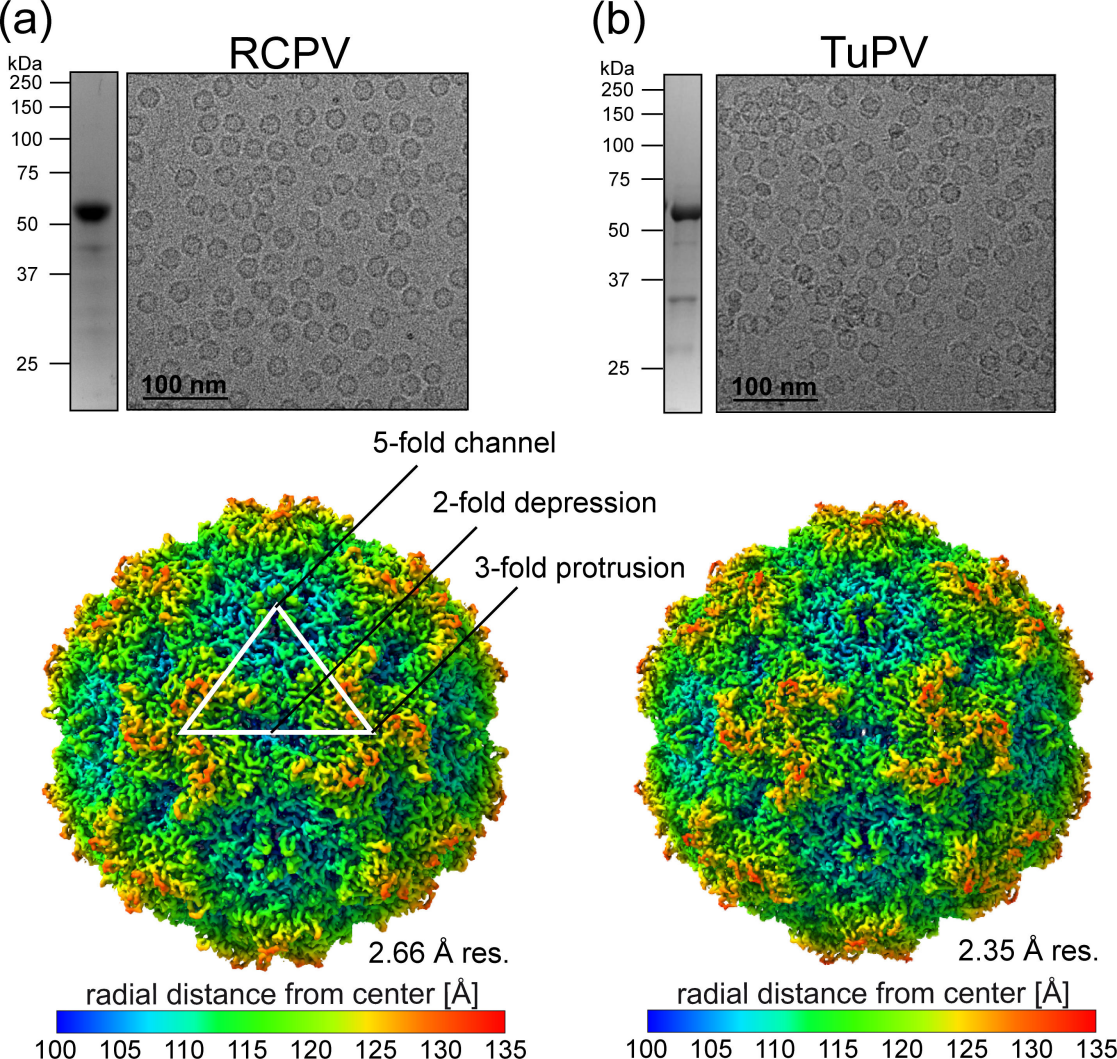
Production/purification and structure determination of RCPV and TuPV. (a) Top panel: an SDS-PAGE of RCPV displaying a band at 60 kDa consistent with the size of VP2 (left side). A cryo-EM micrograph of the same purified sample exhibiting intact capsids that are ~25 nm in diameter. Bottom panel: the capsid surface density map of RCPV determined via cryo-EM reconstruction, which was contoured at a sigma (σ) threshold = 2. The resolution of the structure is calculated based on an FSC threshold = 0.143. The surface density map is radially colored (blue to red) based on the distance to the center of the particle, as shown in the scale bar below. The icosahedral two-, three-, and five-fold symmetry axes are indicated on the RCPV capsid map. (b) Depiction as in panel (a) for TuPV.

To expand the architectural repertoire for the parvoviruses, two aveparvoviruses affecting red-crowned cranes (RCPV) or turkeys (TuPV) were selected for structural determination via cryo-EM representing two species of the genus with ~55% amino acid sequence identity comparing the VP2. The ORFs of these two viruses coding for the major capsid protein VP2 were cloned into the pFastBac1 plasmid for the production of RCPV and TuPV virus-like particles (VLPs), utilizing the standard Bac-to-Bac expression system in insect *Sf9* cells. After the production and purification of the VLPs, both samples were analyzed for purity and concentration using SDS-PAGE. For both viruses, a distinct band consistent for VP2, of approximately 60 kDa, was observed (Fig. 1a). Additional analyses with negative stain electron microscopy (EM) and by cryo-EM showed the presence of intact capsids around 25 nm in diameter. Therefore, the samples were considered suitable to proceed with cryo-EM for high-resolution data collection.

### The RCPV and TuPV capsid structures exhibit short threefold protrusions

Following cryo-EM data collection, three-dimensional reconstructions of both aveparvoviruses, utilizing 405,850 RCPV and 35,131 TuPV extracted individual particles, were determined at resolutions of 2.66 Å and 2.35 Å, respectively (Table 1). Like all other members of *Parvoviridae*, the reconstructed capsids exhibited a channel at the five-fold symmetry axes and depressions at the two-fold axis and surrounding the five-fold channel (Fig. 1b). However, compared to other genera of the subfamily *Parvovirinae* with determined structures, the threefold protrusions appeared less “spiky” and smoother. Similar to tusavirus (TuV), a protoparvovirus, the threefold protrusions of RCPV and TuPV are partially fused into a pinwheel shape (39).

**TABLE 1:** Summary of cryo-EM data collection, image processing, and refinement statistics

Parameter	RCPV	TuPV
Total no. of particles	405,850	35,131
Total no. of micrographs	5,067	562
Defocus range (µm)	0.47–1.90	0.95–1.99
Electron dose (e^−^/Å^2^)	50	50
Pixel size (Å/pixel)	0.72	0.72
No. of frames/micrograph	40	40
Resolution of final map (Å)	2.66	2.35
PHENIX model refinement statistics		
Map correlation coefficients	0.831	0.876
RMSD (root mean square deviation) bonds (Å)	0.01	0.01
RMSD angles (°)	0.81	0.86
All-atom clash score	13.23	13.82
MolProbity score	2.20	1.81
Model resolution (Å)		
FSC_0.143_ (masked)^*a*^	2.66	2.39
FSC_0.143_ (unmasked)	2.73	2.42
Ramachandran plot (%)		
Favored	97.4	98.6
Allowed	2.6	1.4
Outliers	0.0	0.0
Rotamer outliers	0.0	0.0
No. of C_β_ deviations	0.0	0.0

^*a*^ FSC, Fourier shell correlation.

**Fig 2:**
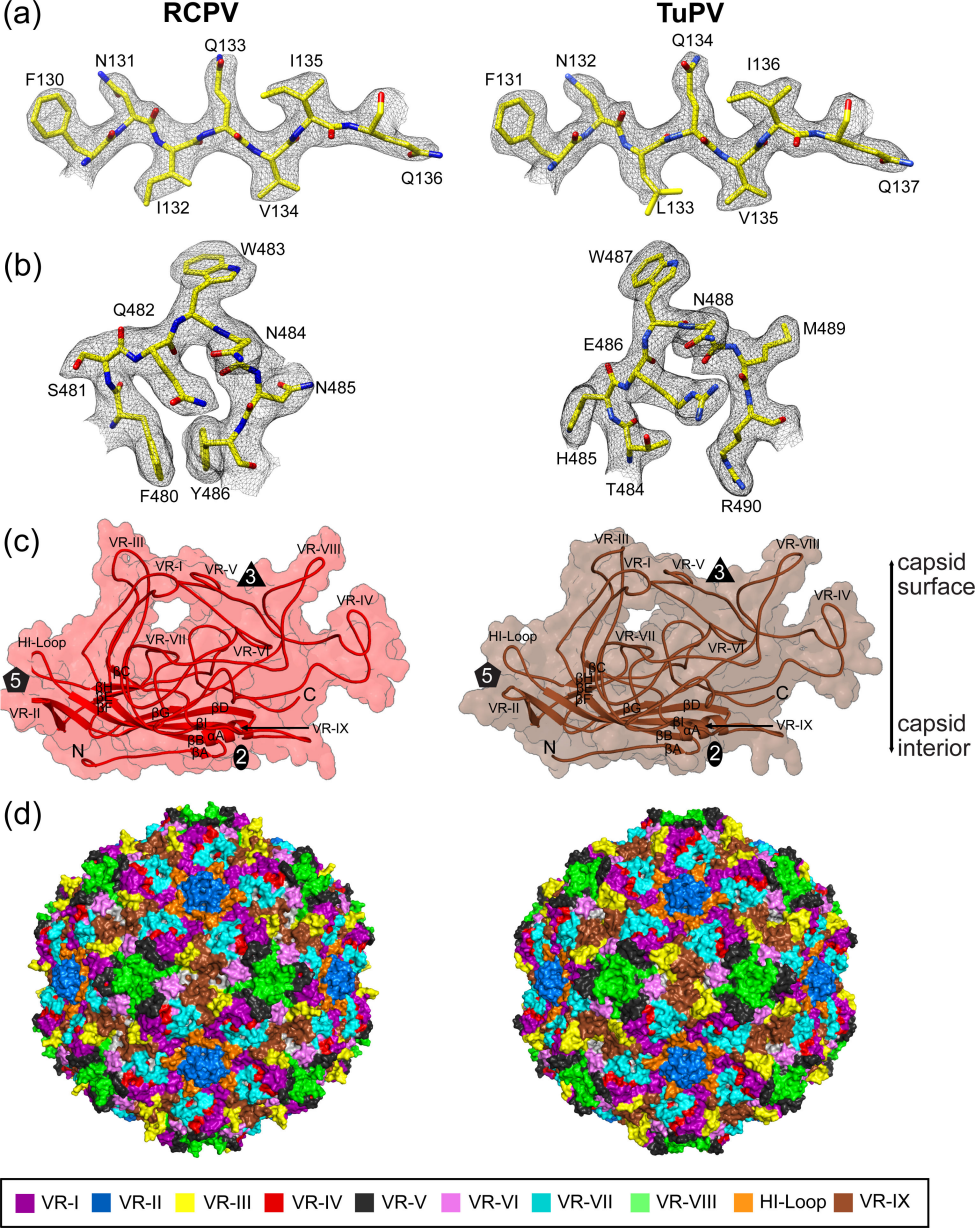
The VP2 monomers of RCPV and TuPV. (a) Seven amino acid residues modeled for β strand in VR-II inside their respective density maps at a sigma (σ) threshold of 2.5 (in black mesh). (b) Seven amino acid residues modeled for VR-IX surface loop inside their respective density maps at a sigma (σ) threshold of 2.5 (in black mesh). All amino acid residues in this figure are labeled and shown as stick representations and colored according to the following color scheme by atom types: C = yellow, O = red, and N = blue. The images were generated utilizing UCSF-Chimera. (c) The VP2 monomer structures are depicted as ribbon diagrams shown inside semi-transparent surface representations. Secondary structure elements, the N- and C-termini, and VRs are labeled. (d) Location of the VRs on the surface of the capsid. The colors are as indicated. These images were generated using PyMOL (41).

Both maps displayed well-ordered side chain densities for amino acid residues forming the core β-strands (Fig. 2a) as well as in the surface loops (Fig. 2b) ranging from aa27 to aa531 (RCPV) and aa30 to aa535 (TuPV). The final atomic models of RCPV and TuPV showed good refinement statistics in addition to high map correlation coefficients (CC) of 0.83 and 0.88, respectively (Table 1). The VP monomer for both aveparvoviruses retained the basic capsid architecture with the conserved eight-stranded jelly roll motif (βB–βI), which is extended by the additional βA-strand and one alpha helix (αA). Between the core secondary structures, loops are inserted that form the surface of the capsids (Fig. 2c). Like other parvovirus capsids, the surface loops are dominated by the large GH loop, located primarily at the threefold region; the DE loop, which forms the fivefold channel pore; and the BC and EF loops, found at the two-/fivefold wall. However, amino acid and structural differences have been observed in these loops, giving unique properties to the individual viruses. Thus, the 10 VRs that have been defined for the parvoviruses can also be adopted for the *Aveparvovirus* genus (Fig. 2d) (Table S1) (34, 40). The short threefold protrusions of the RCPV and TuPV capsids are primarily formed by VR-IV and VR-VIII. The VR-VIII loop of TuPV contains an insertion of a single amino acid relative to RCPV, whereas the VR-V loops have identical lengths in either virus. Another region of divergence is VR-III or EF-loop, where TuPV has a two-residue insertion compared to RCPV, which is located at the two-/fivefold wall. While the amino acid sequence identity is low in these surface loops, the overall rounded and smooth threefold protrusion may play a role in the host specificity toward avian hosts, and regions with higher divergence contribute to the tropism to the specific host and/or tissue selectivity.

### Bivalent cation coordination sites at threefold protrusions of RCPV and TuPV

**Fig 3:**
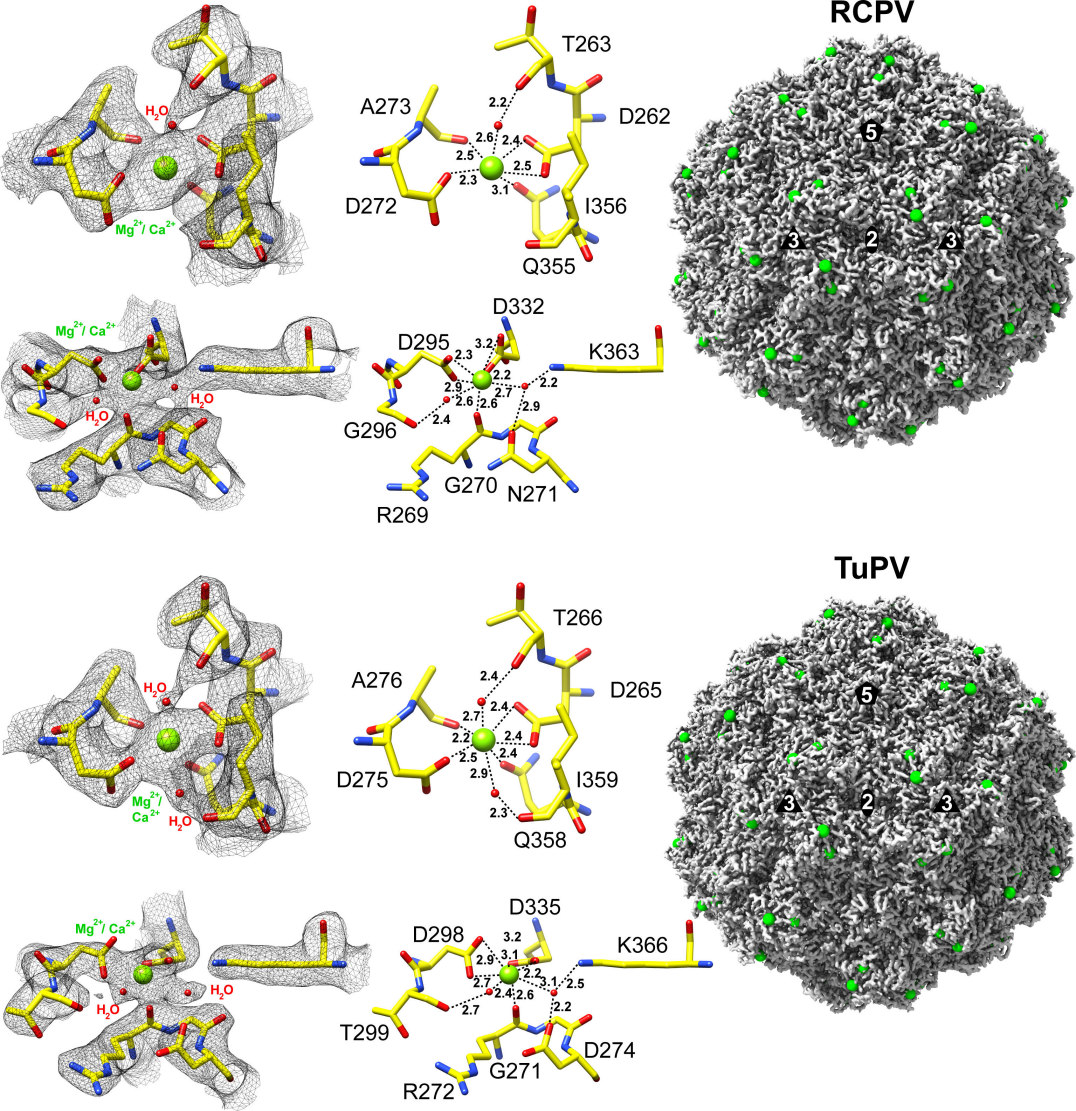
Bivalent cation interaction sites on the RCPV and TuPV VLPs. The amino acid residues are labeled and shown as stick representations and colored according to the following color scheme based on atom types: C = yellow, O = red, and N = blue. Bivalent cation (Ca^2+^ or Mg^2+^) is shown as a sphere and colored in light green. Water molecules are represented as a sphere and colored in red. The images were generated with UCSF-Chimera.

With the technological advancements in recent years, cryo-EM is capable of generating high-resolution maps of macromolecules that allow us to discern fine structural details, such as water molecules or bound metal ions. Both RCPV and TuPV maps had conserved additionally ordered densities in the threefold region of the capsid, which represented potential metal-bivalent cation-binding sites (most likely Mg^2+^ or Ca^2+^) with ordered water molecules (Fig. 3). One is located near the apex of the threefold protrusion, whereas the other one is partially buried at the side of the threefold protrusion. The bivalent cation at the side of the threefold protrusion is coordinated by identical amino acids in RCPV and TuPV, including D262, T263, D272, and A273 (RCPV numbering) of VR-IV and Q355 of VR-VII. Additionally, connected densities to residues too far away for hydrogen bonding were observed and interpreted as ordered water molecules that may contribute to the coordination of the cation. For TuPV, two water molecules were observed at this site, due to the higher resolution of the density map. The second cation site was coordinated by R269, N271 of VR-IV, D295 of VR-V, and D332 of VR-VI. Similarly to TuPV, connected densities to residues too distant for hydrogen bonding (G296 and K363) were found at the first binding site for RCPV and was also interpreted as ordered water molecules. In contrast to the first site, two amino acid differences were observed between RCPV and TuPV (G296T and N271D) for the second binding site, which, however, did not alter the coordination of the cation.

Bivalent cations are most often coordinated by acidic amino acid side chains by binding to the carboxyl side chains found in amino acids such as aspartic acid or glutamic acid (42). All measured distances from the amino acid residues to the bivalent cation are within approximately 2.2–3.2 Å. While magnesium has a typical Mg^2+^-oxygen (O) distance of approximately 2.1 Å, the distance of Ca^2+^-O is in the range of 2.4–2.5 Å (43). Therefore, the bivalent cation is likely a Ca^2+^ ion. Metal ions are prevalent in many macromolecules as they are often critical for proteins to maintain structural stability (44). CPV was previously described with Ca^2+^ sites detected in its capsid, which could contain up to three cations per subunit (44, 45). These potential interactions could occur when the viral proteins associate with ions available in the host cells and are often essential to maintain structural stability and successful genome ejection (46). While the surface loops of RCPV and TuPV generally display large amino acid differences, the cation-binding sites of these viruses are highly conserved, indicating a potential common function. However, to date, little is known about the specific roles these ions play during infection (45).

### RCPV binds to terminal sialic acids

For cell entry, capsids recognize specific receptors on the surface of the host cell. For parvoviruses, the binding of different glycan receptors has been reported. The capsids of viruses from multiple genera were shown to be capable of binding terminal sialic acids including CPV, minute virus of mice (MVM), cutavirus (CuV), TuV, bufavirus, H-1 parvovirus, and LuIII of the protoparvoviruses, bovine parvovirus (BPV) of the bocaparvoviruses, and AAV1, AAV4, AAV5, AAV6, and serpentine AAV (SAAV) of the dependoparvoviruses (39, 47–52). However, several parvoviruses require specific glycosylation linkages for the binding to the sialic acids. Among the α2-3 sialic acids binding parvoviruses are AAV1, AAV4, AAV5, AAV6, SAAV, BPV, TuV, MVM, H-1PV, LuIII, CPV, feline panleukopenia virus (FPV), and porcine parvovirus (PPV), whereas α2-6 sialic acid binders include AAV1, AAV5, AAV6, SAAV, CuV, H-1PV, LuIII, CPV, FPV, and PPV (39, 48, 50, 52–54). Additionally, some parvoviruses also bind to α2-8 linked sialic acids such as MVM and TuV (39, 53). On an additional level, these glycans can be attached either to proteins via asparagines (N-glycans) and threonine/serines (O-glycans) or to lipids (gangliosides), which further increases the glycan variability on cell surfaces to which the parvoviral capsids need to adapt to enable binding with their capsids (53).

There have been several structural studies utilizing either x-ray crystallography or cryo-EM to determine the binding sites of sialic acids to several parvovirus capsids. Based on these structural studies, it was observed that the sialic acids bind to different capsid regions for different parvoviruses. For AAV1 and AAV6, density indicating the presence of sialic acid was detected in a pocket located at the bases of the protrusions at the icosahedral threefold symmetry axes near the two-/five-fold wall (50). In contrast, AAV5 binds sialic acid near the center of the threefold protrusions and SAAV at the two-/five-fold wall (52, 55). Among the protoparvoviruses, the sialic acid binding sites were found near the twofold depression for the MVM, LuIII, and H-1PV capsids (48, 54).

**Fig 4:**
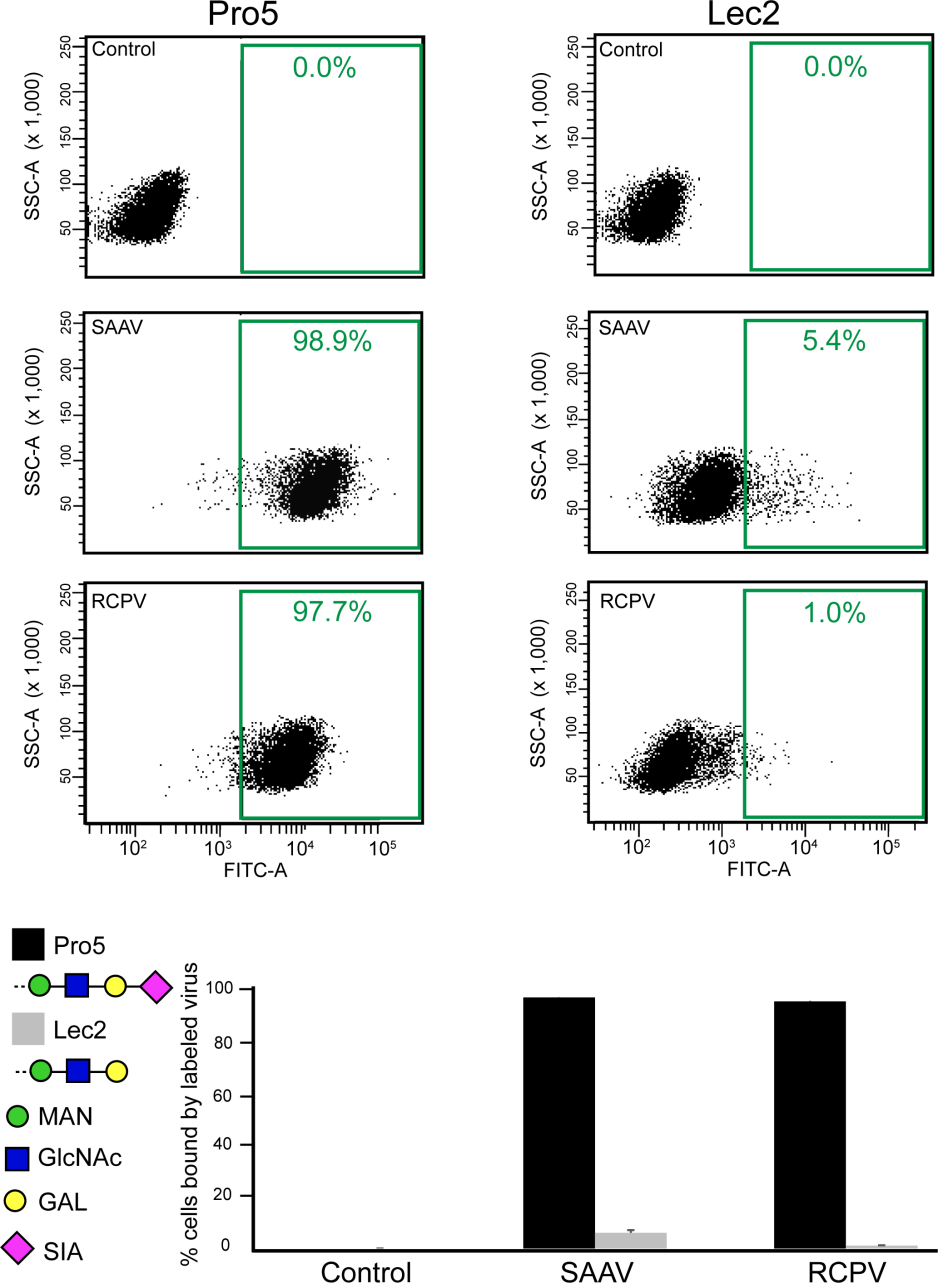
RCPV is a sialic acid binder. Fluorescent-labeled VLPs were incubated with CHO Pro5 and Lec2 cell lines that display terminal sialic acid or galactose, respectively. The binding of the VLPs was determined via an FACS-based assay. Shown are example FACS histograms for control cells and cells incubated with RCPV and SAAV, and the percentages of bound cells are provided. Below is a summary of the triplicate experiments. The surface glycans of the cell lines are represented as symbols.

To determine whether RCPV binds to sialic acid to attach to the cell, a cell-binding assay using differential glycan-presenting variant Chinese Hamster Ovary (CHO) cell lines was conducted. While CHO-Pro5 displays terminal sialic acid, CHO-Lec2 displays terminal galactose, differing only by a deletion mutation of the CMP-sialic transporter gene, resulting in decreased levels of terminal sialic acids on the cell surface (56). The SAAV capsid is known to strongly recognize terminal sialic acids as a receptor and was utilized as a positive control that bound to Pro5 in the cell binding assay (52). To determine the sialic acid binding capacity of RCPV, fluorescently labeled RCPV and SAAV capsids were analyzed by a fluorescence-activated cell sorting (FACS)-based detector. RCPV showed robust binding to the Pro5 cells but not to the sialic acid-deficient cell line Lec2, displaying a similar binding pattern to SAAV (Fig. 4). Thus, *Aveparvovirus* is the fourth genus with at least one member shown to bind sialic acid. Future studies will need to determine the specific sialic acid linkage required by RCPV and whether they represent N- or O-glycans. Most birds express α2-3-linked sialic acids in their tissues; however, depending on the exact species, α2-6-linked sialic acids are found in the respiratory tract or intestines (57). Additionally, it remains to be established whether sialic acid is utilized as a receptor *in vivo*, acts only as an attachment factor, where its binding site is located on the RCPV capsid, and whether other members of this genus also bind to sialic acid.

### Comparison of RCPV and TuPV monomers to AlphaFold 3 generated predictions

**Fig 5:**
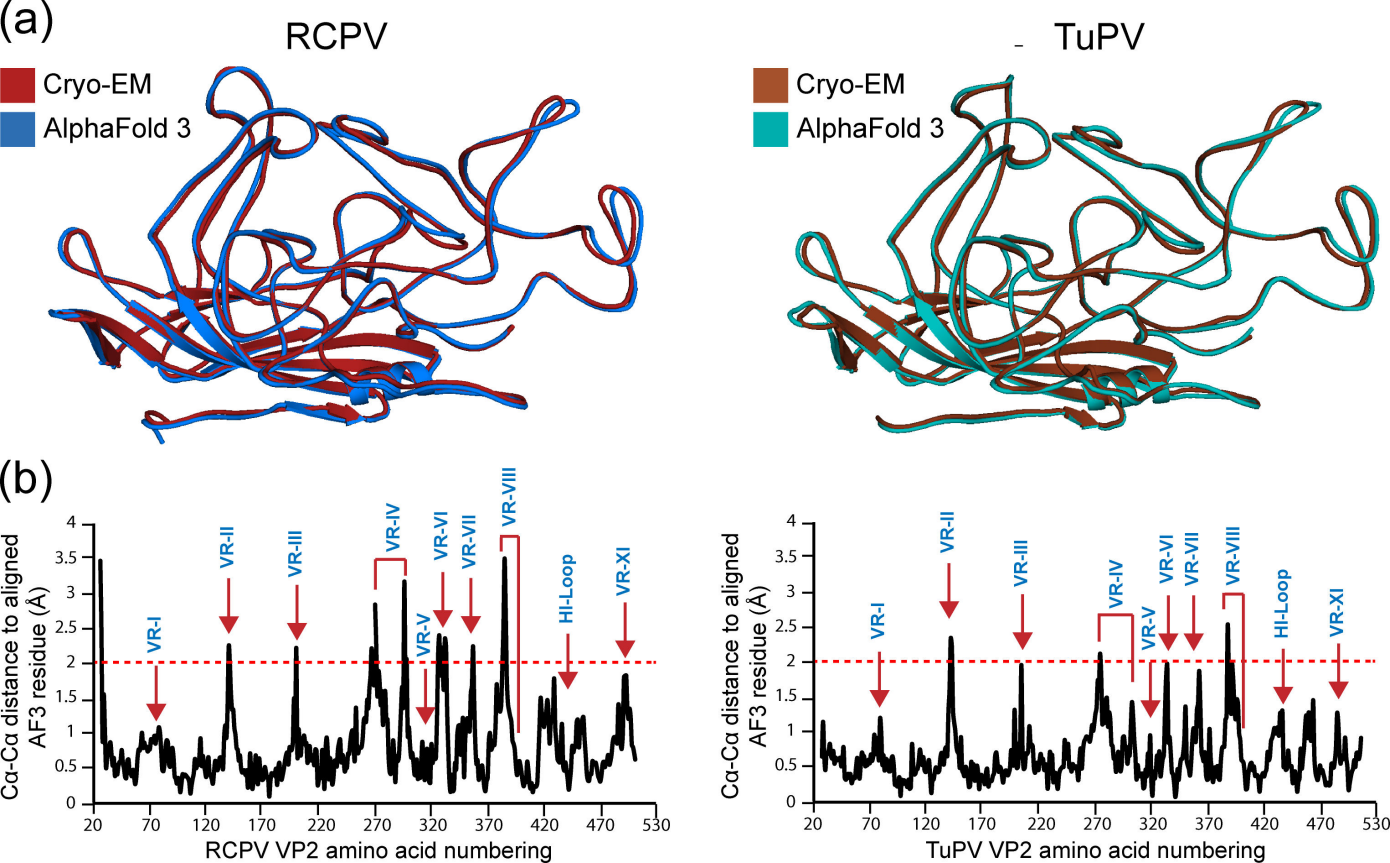
Comparison of AlphaFold 3 predictions against cryo-EM determined VP structures of RCPV and TuPV. (a) Structural superposition of RCPV (left) and TuPV (right) determined by cryo-EM and AlphaFold 3 is shown as ribbon diagrams. The images were generated in PyMOL (41). (b) The Cα-Cα distance plot for the RCPV (left) and TuPV (right) amino acid residues relative to the AlphaFold 3 predicted structures when the VP structures are superposed. The assigned variable regions are labeled in blue.

The introduction of AlphaFold (AF; current version AF3) has fueled a breakthrough in modeling protein structures and their interactions. Since its inception, AF has been shown to be capable of high-accuracy protein structure prediction (58). However, AF3 is trained upon structures that are readily available in the Protein Data Bank (PDB). Prior to this study, no aveparvovirus structures were available in the PDB, which could potentially pose a limitation to protein structure prediction. To test the accuracy of AF3, RCPV and TuPV monomer protein predictions were generated utilizing AF3. Based on the predicted local distance difference test (pLDDT), the predicted structures of RCPV (Fig. S1a) and TuPV (Fig. S1b) are of high confidence, with the exceptions of VR-III, VR-V, VR-VI, and the N-terminus, which is expected to be intrinsically disordered. The AF3 predictions were then compared to their respective structures determined via cryo-EM (Fig. 5a). After the monomers were superposed, the conserved jelly-roll motif aligned nearly perfectly with Cα-RMSDs of less than 0.4 Å, which was not surprising, as this is the most conserved feature among all parvoviruses. However, in the surface loops, minor disparities between the AF3 and cryo-EM structures were observed with Cα-Cα distances up to 3.5 Å for RCPV and 2.5 Å for TuPV at the VR-VIII loop apices (Fig. 5b). The most notable disparities between the generated prediction and experimental structure are VR-IV and VR-VIII. Nonetheless, AF3 is a valuable tool to generate a starting model for further refinement using cryo-EM and the general structure prediction of new viruses.

### Structure predictions of other members in the *Aveparvovirus* genus

**Fig 6:**
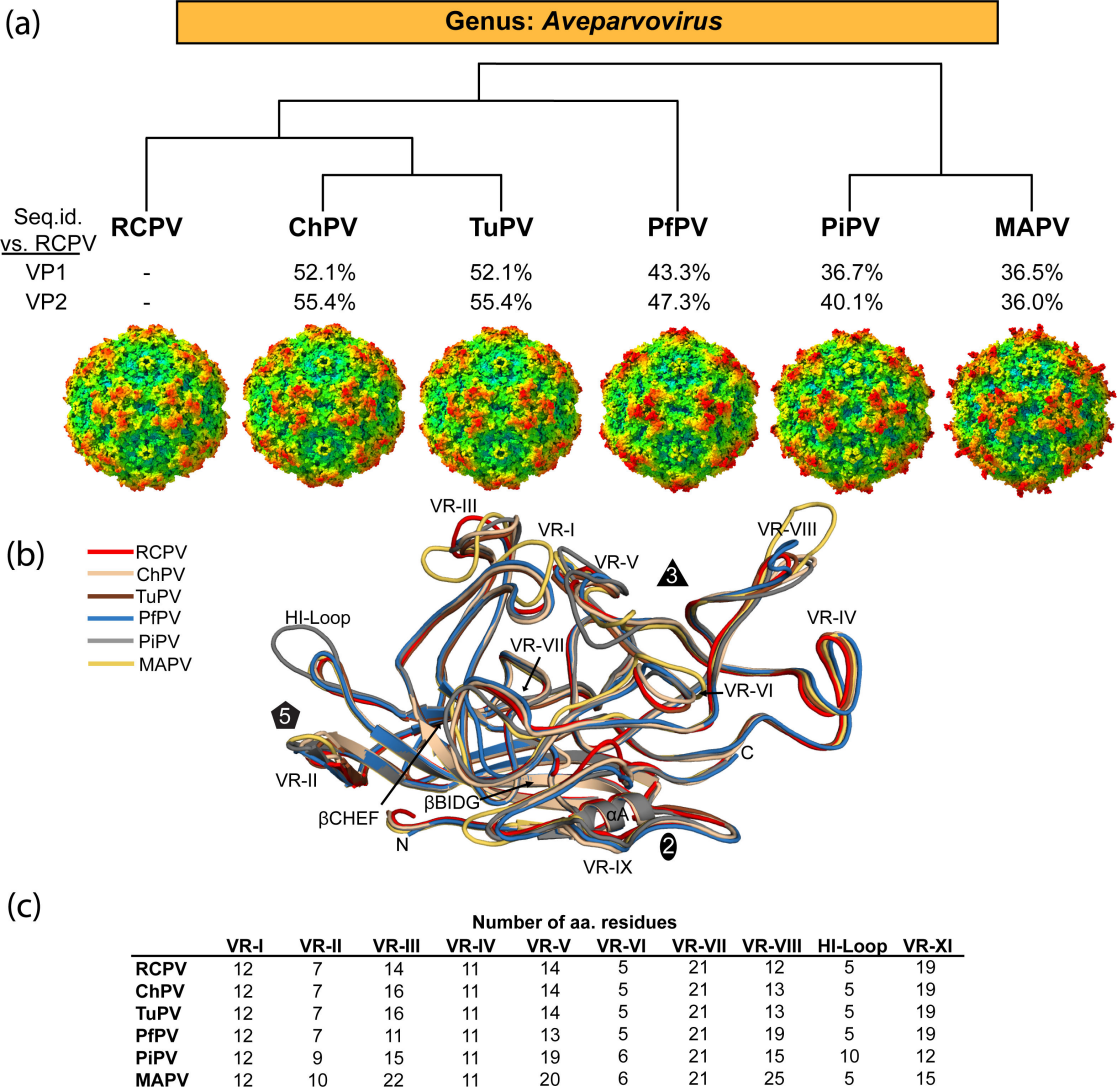
The *Aveparvovirus* genus. (a) A phylogenetic tree of all *Aveparvovirus* members was generated online (ngphylogeny.fr) utilizing the VP2 amino acid sequences as the input (62). Amino acid sequence identities were compared for the VP1 and VP2 of all aveparvoviruses vs RCPV. (b) A comparison of AlphaFold 3 generated surface representations of ChPV, PfPV, PiPV, and MAPV to RCPV and TuPV is shown. The superposition of the aveparvoviruses VP structures is depicted as cartoon ribbon diagrams below. The VRs, β-strands, and the icosahedral symmetry axes are labeled. (c) Table summarizing the number of amino acid residues for each of the VRs for all aveparvoviruses.

Hence, AF3 was used to predict the capsid structures for the other four members of the *Aveparvovirus* genus: ChPV, PfPV, PiPV, and MAPV (58). A phylogenetic analysis based on the VP2 amino acid sequences shows that TuPV and ChPV share the closest identity, with MAPV being the most distant member (Fig. 6a). Compared to RCPV, the amino acid sequence identity based on VP1 and VP2 is the lowest for MAPV at 36.5% and 36.0%, respectively. For all the aveparvoviruses, the aa sequence identity for VP1 is within 36.5%–52.1% and for VP2 is within a comparable range of 36.0%–55.4% (Fig. 6b). Despite the low sequence identity, there are structural similarities between RCPV, TuPV, and ChPV capsids. When superposed, the surface loops of these aveparvoviruses are nearly perfectly overlaid, with only minor differences in most surface loops, with the exception of VR-III where both ChPV and TuPV adopt a different loop conformation than RCPV due to a 2 aa insertion. More significant differences were also observed when the RCPV structure was superposed against the PfPV, PiPV, and MAPV models, and the lengths of the VRs were determined for all the aveparoviruses (Fig. 6c). The most distinct differences for PfPV were found in VR-III and VR-VIII with a 3 aa deletion and a 7 aa insertion (vs RCPV), respectively. For PiPV, different loop conformations were observed for VR-II (2 aa insertion), -III (1 aa insertion), -V (5 aa insertion), -VI (2 aa deletion), -VII (same loop length), -VIII (3 aa insertion), and the HI loop (5 aa insertion). Lastly, for MAPV, differences in loop conformations were found in VR-II (3 aa insertion), -III (8 aa insertion), -V (6 aa insertion), -VI (2 aa insertion), -VIII (13 aa insertion), and -IX (4 aa deletion). The insertions primarily in VR-V and -VIII affect the appearance of the threefold protrusions. As a result, the threefold protrusions of PfPV radiate further outward near the threefold axis and for PiPV and MAPV further away from the threefold axis. Differences in the threefold region could contribute to diverse epitopes for neutralizing antibodies and host cell receptor binding sites in their respective avian hosts (59). The HI loop is longer for PiPV than for the other members, affecting the appearance of the region surrounding the fivefold channel. Prior mutagenesis studies in other parvoviruses suggest that this region plays a critical role in genome packaging and ejection (60, 61).

### Structural comparison of RCPV to published structures of other genera in *Parvovirinae*

**Fig 7:**
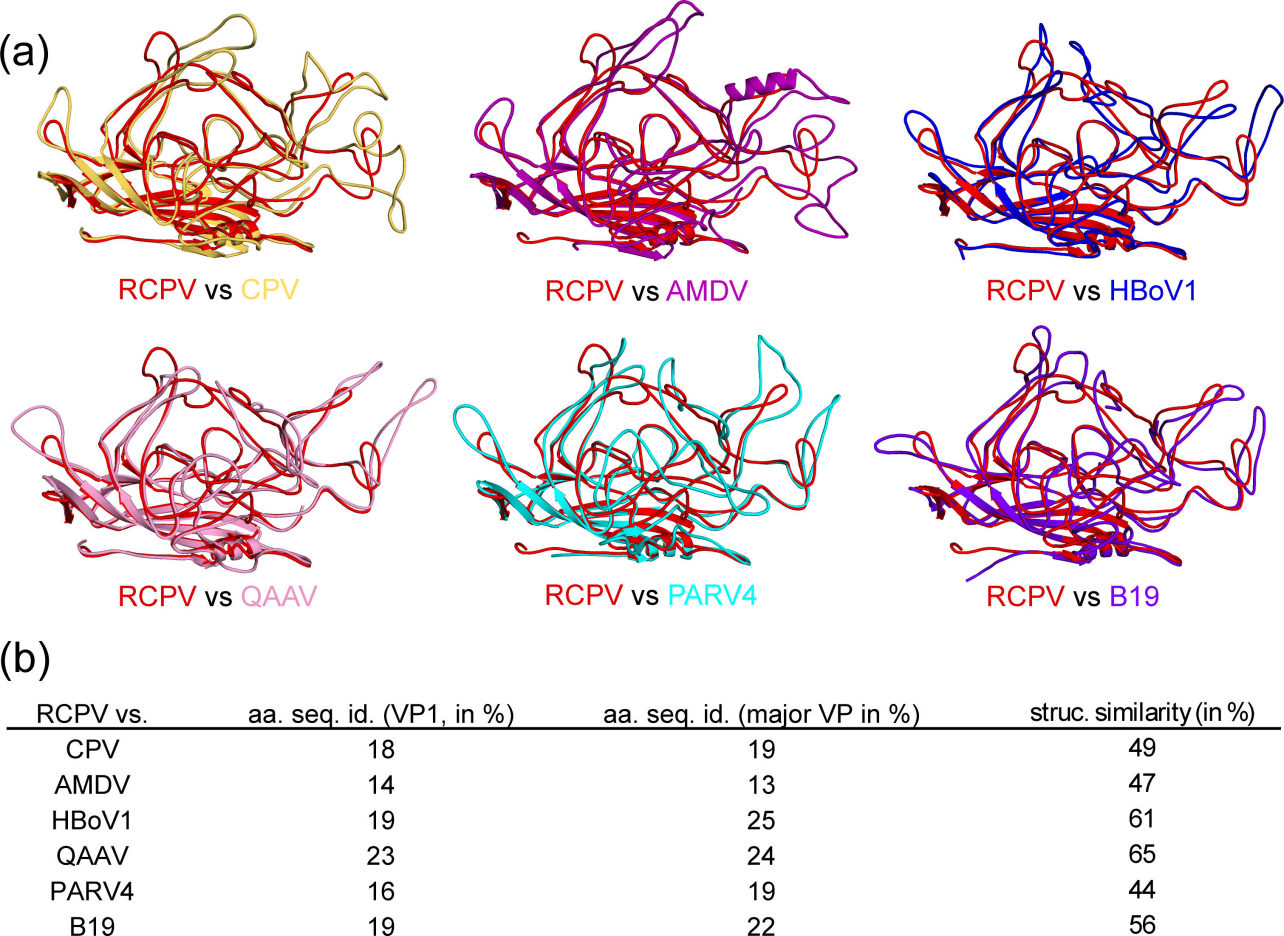
Comparison of RCPV VP to VPs from other genera in *Parvovirinae*. (a) The major VP monomer structures are depicted as ribbon diagrams for RCPV against a representative member of six other *Parvovirinae* genera. (b) Table of Cα distances of major VP and amino acid sequence identity of VP1 and major VP of RCPV compared to CPV, AMDV, HBoV1, QAAV, PARV4, and B19.

RCPV and TuPV are two new additions to the structural portfolio of the subfamily *Parvovirinae* and were superposed against CPV, Aleutian mink disease virus (AMDV), human bocavirus 1 (HBoV1), quail AAV (QAAV), human parvovirus 4 (PARV4), and parvovirus B19 as a structural comparison to other genera (Fig. 7a) (32, 63–65). The capsid core is almost perfectly superposable when compared to members from other *Parvovirinae* genera. However, the vast majority of structural differences are found in most of the VRs of the capsids. Significant structural differences were found in all surface loops. For example, VR-IV was significantly shorter for RCPV than all other genera in *Parvovirinae*. These differences contribute to the truncated appearance of the threefold protrusions and fivefold pores of RCPV. RCPV is most structurally similar to QAAV, with a structural similarity of 65%, a *Dependoparvovirus* which affects bob-white quails. On the other extreme, RCPV shares the least structural similarity to PARV4, a *Tetraparvovirus* which was first reported in a hepatitis B virus-infected injecting drug user in 2005 (66). Because RCPV and QAAV both affect avian hosts, the host could dictate the structural design of the viruses.

The VP1 of RCPV possesses an aa sequence identity of 18%, 14%, 19%, 23%, 16%, and 19% to CPV, AMDV, HBoV1, QAAV, PARV4, and B19, respectively. Similarly, for VP2, the RCPV has an aa sequence identity profile within 13%–25% (Fig. 7b). As the capsid core is highly conserved among all parvoviruses, the vast majority of the amino acid differences are located in the surface loops. These differences in aa sequence and structural identity have been associated in the past with antigenicity and cellular transduction in parvoviruses, which could suggest that RCPV has alternative tropism and immunogenicity.

## Conclusions

The first high-resolution structures of RCPV and TuPV are presented. Both aveparvoviruses share the same *T* = 1 icosahedral capsid architecture as other parvoviruses, conserving the capsid’s fundamental functions for receptor recognition, capsid assembly, as well as genome replication, packaging, and release. However, the aa acid sequences and surface loop conformations of RCPV and TuPV are shown to be substantially different compared to members of other genera. These variations in the capsids could be associated with host-specific receptor attachment and antigenic diversification. Moreover, the identification of sialic acid—a common glycan utilized by other parvoviruses—as a potential receptor for RCPV provides insight into cell attachment and entry.

Compared to the previously well-characterized genera of *Parvovirinae*, the aveparvoviruses are still poorly understood. Therefore, the capsid structures presented in this manuscript will aid in the future characterization of these pathogens and toward the development of vaccines or antivirals.

## MATERIALS AND METHODS

### Expression and purification of virus-like particles

VLPs of RCPV and TuPV were expressed using the Bac-to-Bac baculovirus system (Invitrogen) as described previously and according to the manufacturer’s instructions (67). The ORFs for VP2 were synthesized (Azenta/Genewiz) and inserted into the pFastbac plasmid (RCPV: nt 3812–5407, accession no. NC_040603; TuPV: nt 3005–4615, accession no. NC_038534). To express VLPs, suspension Sf9 insect cells were cultured in Sf-900 SFM media to a confluency of 1.5–2.5 × 10^6^ cells/mL and infected with recombinant baculoviruses at a multiplicity of infection (MOI) of 10. After 72 h post-infection, the cells were harvested and pelleted by centrifugation at 1,000 × *g* for 10 min at 4°C. The Sf9 cell pellets were resuspended in 1× TNTM buffer (25 mM Tris-HCl, 100 mM NaCl, 0.2% Triton X-100, and 2 mM MgCl_2_, pH 8.0). The cell pellets underwent three freeze-thaw cycles and were benzonase-treated (125 U/mL) for 1 h at 37°C. After clarification by centrifugation at 12,000 × *g* for 30 min to remove cellular debris, the resulting clarified supernatant was loaded onto a 20% sucrose cushion ([wt/vol] in 1× TNTM buffer) and centrifuged at 45,000 rpm in a Ti70 rotor for 3 h at 4°C. The pellet was resuspended in 1× TNTM buffer. For purification, the resuspended pellet was loaded onto a 10%–40% sucrose step gradient ([wt/vol] sucrose in 1× TNTM buffer) and centrifuged using a SW41 rotor for 3 h at 4°C. Individual fractions were collected from the gradient and analyzed by SDS-PAGE. Fractions showing the expected VP2 band at ~60 kDa were dialyzed in phosphate-buffered saline (PBS) and further concentrated to >0.1 mg/mL using centrifugal filter concentrators (Sigma Aldrich). The purified samples were stored long-term at −80°C.

### RCPV and TuPV sample purity and integrity

The purity and integrity of the samples were confirmed by SDS-PAGE and negative-stain EM, respectively. For the SDS-PAGE analysis, the samples were incubated with 1× Laemmli Sample Buffer (Bio-Rad) with 10% (vol/vol) β-mercaptoethanol. The samples were boiled for 10 min at 100°C, and then the denatured proteins were loaded onto a 10% SDS-PAGE and run at 80 V. After the run, the gel was washed three times with distilled water (diH_2_O) and stained with GelCode Blue Protein Safe stain (Invitrogen) for 1 h. After staining, the gel was destained and washed three times with diH_2_O and imaged using a GelDoc EX system (Bio-Rad). Based on the SDS-PAGE, the purified protein yield for RCPV and TuPV was approximately 0.4 µg/mL culture and 0.08 µg/mL culture, respectively. For the negative stain EM, 5 µL of each sample was incubated onto glow-discharged CF400-CU carbon-coated 400 mesh copper grids (Electron Microscopy Sciences) for 2 min and washed in three 15 µL droplets of nuclease-free water (Thermo Fisher Scientific). The excess water was blotted with filter paper (Whatman), and the grid was stained with filtered 2% uranyl acetate. Excess stain was blotted, and the grids were analyzed and imaged utilizing a Tecnai G2 Spirit Transmission Electron Microscope (FEI) operating at 120 kV.

### Vitrification and cryo-EM data collection

Three microliter of the purified RCPV and TuPV VLPs was applied to glow-discharged C-flat holey carbon-coated grids (Protochips Inc.) and was vitrified via the Vitrobot Mark IV (FEI) automatic plunge-freezing system. The samplers were both incubated on grids at 4°C and 95% humidity for 3.0 s prior to blotting with filter paper, followed by plunging into a liquid ethane bath for vitrification. Until data collection, the grids were maintained at liquid nitrogen temperatures. To screen the particle distribution and ice quality of the grids, a FEI Tecnai G2 F20-TWIN microscope (FEI Co.) was utilized in-house under low-dose conditions (200 kV, ~20e^−^/Å^2^). After the screening process, cryo-EM data collection was conducted at the Structural Biology Cryo-Electron Microscopy Core at The Herbert Wertheim UF Scripps Institute for Biomedical Innovation and Technology using the JEOL CryoARM300. The JEOL CryoARM300 was operated at 300 kV with energy filtering, and the data were collected on a Gatan K3 detector with the Omega filter slit width at 20 eV. During the data collection, a total dose of 50 e^−^/Å^2^ was used for 50 movie frames per micrograph. All movie frames were aligned utilizing Patch Motion Corr with dose weighting (68).

### 3D particle reconstruction

The software package cisTEM was utilized for 3D image reconstruction of the electron density maps for RCPV and TuPV, as reported previously (Fig. S2a) (69). The aligned micrographs were first imported, and the microscope-based contrast transfer function was estimated. Suboptimal-quality micrographs in the data set were discarded, and particles were automatically picked using a characteristic particle radius of 125 Å. Following particle picking, the individual capsid images were sorted into 20 classes using 2D classification (Fig. S2b and C). Any classes that contained impurities were removed. An initial map was generated using the Ab-Initio 3D function and was further refined using the automatic refinement function with default settings. For the final electron density maps, the maps were sharpened using the pre-cutoff *B*-factor value of −90 Å^2^ and variable post-cutoff *B*-factor values of 0, 20, and 50 Å^2^. In UCSF-Chimera, the sharpened electron density maps were analyzed, and the −90 Å^2^/0 Å^2^ map was utilized for model building and structure refinement. The resolutions of the maps were estimated at a Fourier shell correlation threshold criterion of 0.143 (Table 1) (Fig. S2b and c).

### Model building and structure refinement

The AlphaFold 3 online server was used to generate *in silico* model predictions of the RCPV and TuPV VP2 monomers, using their primary amino acid sequences (58). The ViperDB oligomer generator was then used to generate a 60-mer (60 copies of VP2) of the models (70). The 60-mer models were docked in their respective cryo-EM density maps using the “Fit in Map” option in UCSF-Chimera. To maximize the CC, the pixel size was optimized to maximize the correlation coefficient. The maps were resized using the EMAN2 subroutine e2proc3d.py based on the best-fit parameters, which were determined by correlation coefficients from UCSF-Chimera. Maps were converted to the CCP4 format via MAPMAN. The main and side chains of the AlphaFold 3 models were manually refined in Coot with the real-space refinement tool. Further refinements of the models in PHENIX provided the final refinement parameters and statistics (Table 1).

### AlphaFold 3 structure prediction

VP models of *Aveparvovirus* members were generated using AlphaFold 3 (https://deepmind.google/science/alphafold/) based on the primary amino acid sequences of RCPV (accession no. YP_009552129), TuPV (accession no. YP_009507348), ChPV (accession no. YP_009046820), PfPV (accession no. YP_010802416), PiPV (accession no. YP_010797213), and MAPV (accession no. QTE03874). The prediction models were colored based on pLDDT, which aids in visualizing the model’s confidence at each amino acid residue with scores ranging from 0 to 100. While higher scores (blue and cyan) indicate greater confidence, lower scores (yellow and orange) indicate that the region is intrinsically disordered or poorly predicted.

### Capsid structure comparison

To structurally compare RCPV and TuPV to members of other genera in *Parvovirinae*, the RCPV and TuPV monomers were superposed in Coot onto the previously resolved monomers for CPV (PDB ID: 2CAS), AMDV (PDB ID: 8EP2), HBoV1 (PDB ID: 5URF), QAAV (PDB ID: 8TEX), PARV4 (PDB ID: 8EP9), and B19 (PDB ID: 1S58). To calculate the percentage of structural similarity for RCPV vs other viruses, the secondary structure matching subroutine in Coot measured the Cα–Cα distances (in Å) between the compared VP structures (71). To determine the percentage of amino acid sequence identity for RCPV vs other viruses, AlignX in VectorNTI was used to compare the VP1 and the VP2/VP3 (major VP) sequences.

### Fluorescent labeling of VLPs

RCPV and SAAV VLPs were fluorescently labeled using the DyLight 488 Antibody Labeling Kit (Thermo Fisher), according to a modified version of the manufacturer’s instructions. A total of 20 µL of borate buffer (0.67 M, pH 8.5) was added to the 25 µL of the purified VLPs, which had concentrations >0.5 mg/mL. Then, the entire volume was transferred to a DyLight Reagent vial. The samples were incubated in light-protected tubes for 1 h at room temperature (RT). After the incubation, 10 µL of borate buffer was added to the reaction and incubated for 30 min at RT. Excess and unbound fluorescent molecules were removed from the samples through three rounds of dialysis in 1× PBS. An SDS-PAGE confirmed the success of the labeling procedures with the presence of fluorescent VP bands of ~60 kDa in size when viewed under UV light.

### Cell binding assay

Pro-5 and Lec-2, CHO variant cell lines, were passed to 50% confluency in 15 cm cell culture plates, 24 h prior to performing the experiment. On the day of the assay, cells at approximately 90%–100% confluence were detached from the surface of the plate by adding 2 mL 0.5 M EDTA to the medium. The cells and media were transferred into a 50 mL conical tube and pelleted via benchtop centrifuge for 5 min at 500 rpm. After removing the supernatant, the cell pellet was resuspended in 5 mL of pre-chilled un-supplemented MEM Alpha media. The cells were counted using a hemocytometer under a microscope, diluted to 5 × 10^5^ cells/mL, aliquoted to 500 µL fractions in Eppendorf tubes, and pre-chilled under constant rotation for 30 min at 4°C. Each aliquot of cells was then incubated with fluorescently labeled VLPs at an MOI of 5 × 10^5^ under constant rotation and protected from light for 3 h at 4°C. After the incubation period, the cells were pelleted at 2,000 rpm for 10 min using a benchtop centrifuge, and the supernatant was discarded. The VLPs that did not bind to the cells were removed by washing the cells with 500 µL pre-chilled 1× PBS buffer. The cells were then pelleted at 2,000 rpm for 10 min in a benchtop centrifuge, with the supernatant discarded afterward. The resulting pellets were resuspended in 500 µL 1× PBS and analyzed using the FACS Canto II Flow Cytometry system (BD Biosciences) in triplicate. To analyze the raw data, the software suite FACSDiva 9.2 (BD Biosciences) was utilized.



## Data Availability

Both density maps reconstructed via cryo-EM and models built for the RCPV and TuPV capsids were deposited in the Electron Microscopy Data Bank (EMDB) under accession numbers EMD-48926 (https://www.ebi.ac.uk/emdb/EMD-48926) and EMD-48927 (https://www.ebi.ac.uk/emdb/EMD-48927) and in the Protein Data Bank (PDB) under 9N5L (https://doi.org/10.2210/pdb9n5l/pdb) and 9N5M (https://doi.org/10.2210/pdb9n5m/pdb), respectively.

## SUPPLEMENTAL MATERIAL

Supplemental material jvi.00110-25-s0001.docx Figures S1 and S2; Table S1.

ASM does not own the copyrights to Supplemental Material that may be linked to, or accessed through, an article. The authors have granted ASM a non-exclusive, world-wide license to publish the Supplemental Material files. Please contact the corresponding author directly for reuse.
