# Supplementary material for: Birds of a feather flock together: structural characterization of red-crowned crane and turkey aveparvoviruses
Source: J Virol. 2025 Jul 3;99(7):e00110-25. doi: 10.1128/jvi.00110-25 (PMC12282197; doi:10.1128/jvi.00110-25)
Supplement: Supplemental material — Figures S1 and S2; Table S1. [file jvi.00110-25-s0001.docx]

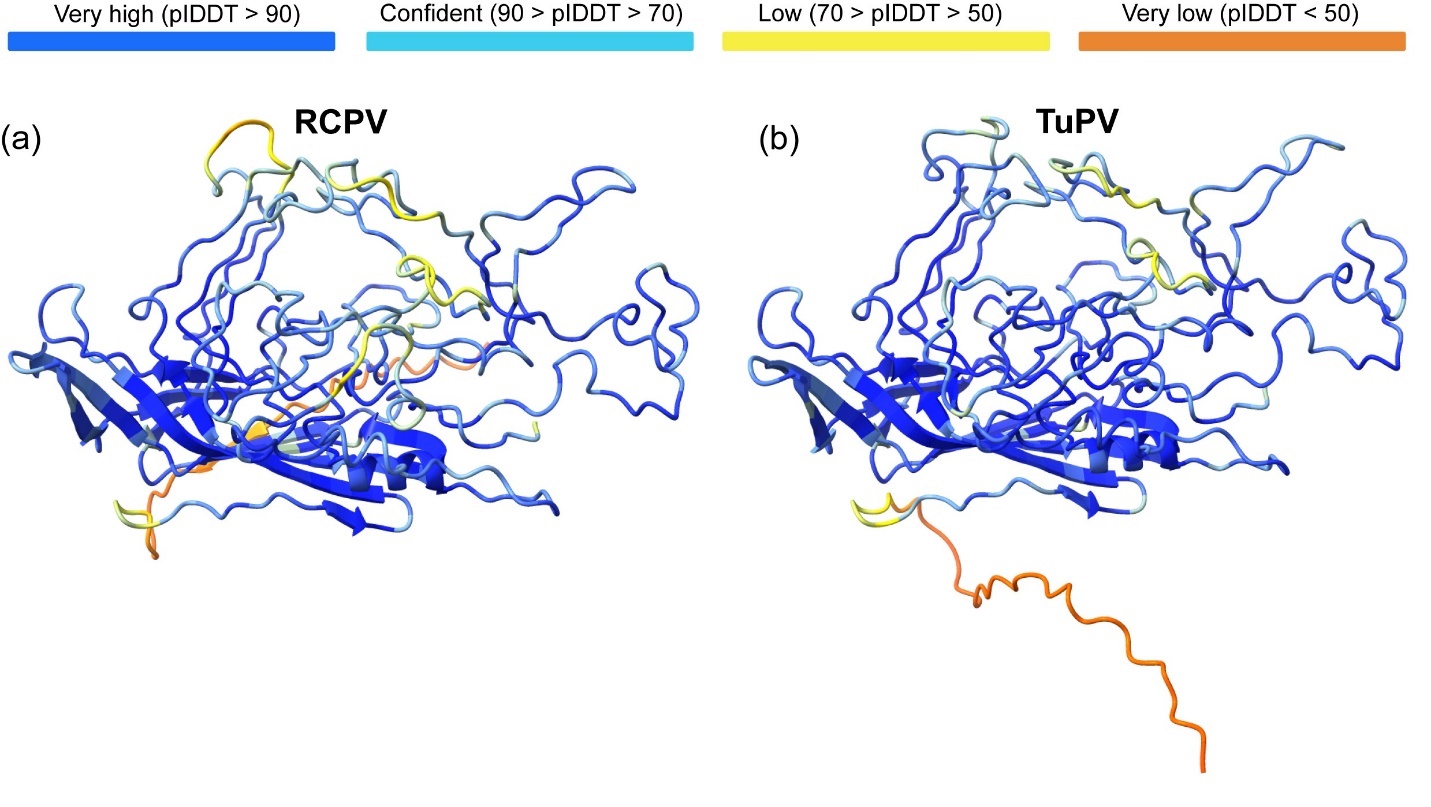


**Supplementary Figure 1.** AlphaFold 3 predictions for RCPV and TuPV capsid monomers. Color range (top) indicates the accuracy of the predicted structures at each residue with plDDT scores ranging from 0 to 100, where higher scores indicate greater confidence for the predictions. **(a)** The plDDT coloring for RCPV and **(b)** TuPV.


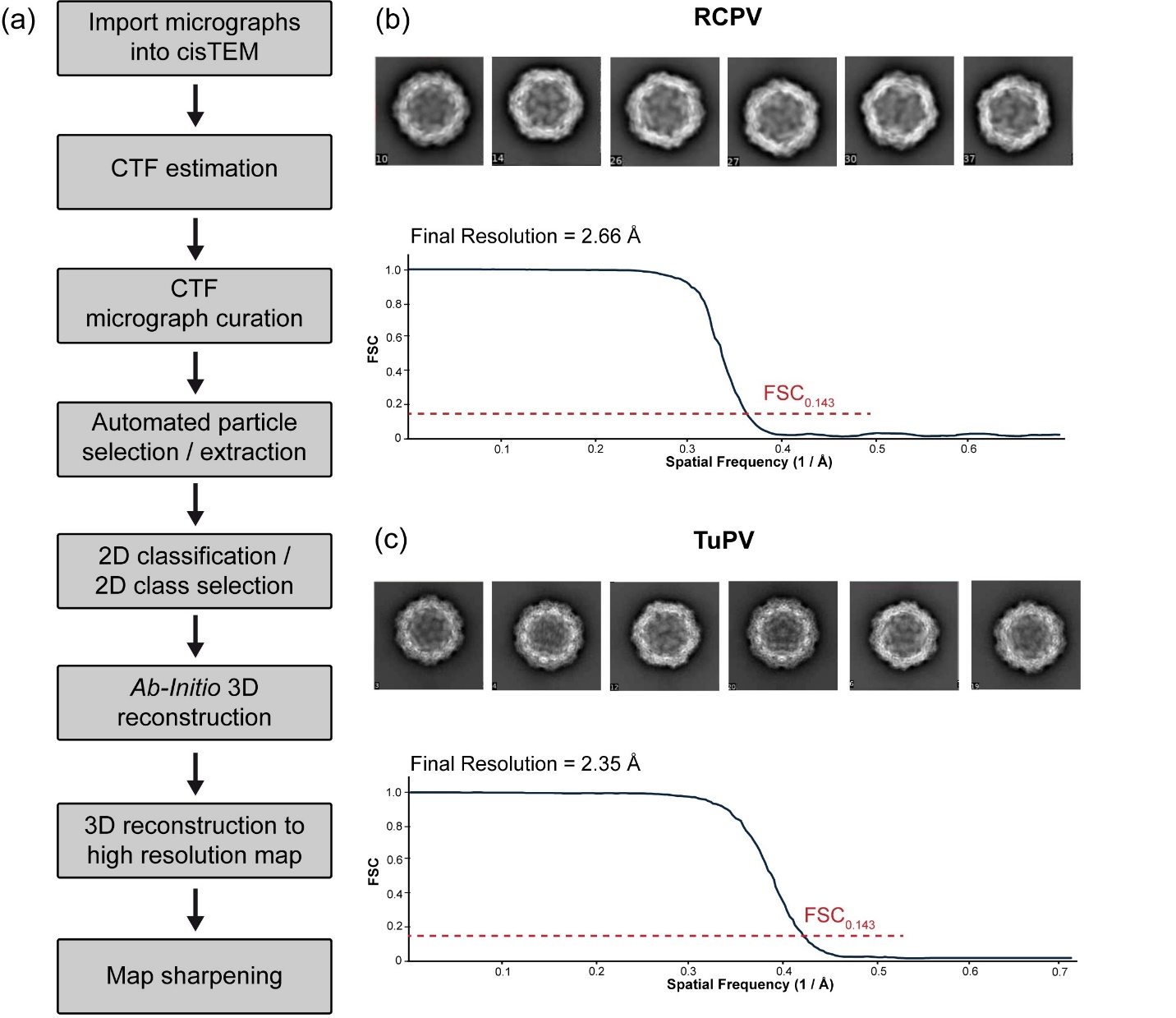


**Supplementary Figure 2.** Overview of cryo-EM data reconstruction in cisTEM. **(a)** A schematic of the general workflow for the processing of the cryo-EM data in the software cisTEM is shown. **(b)** The top panel shows representative 2D classes for RCPV. Below the FSC curve for the final map is provided. **(c)** Same depiction as in panel (b) except for TuPV.

|  | **VR-I** | **VR-II** | **VR-III** | **VR-IV** | **VR-V** | **VR-VI** | **VR-VII** | **VR-VIII** | **HI-Loop** | **VR-IX** |
| --- | --- | --- | --- | --- | --- | --- | --- | --- | --- | --- |
| **RCPV** | 72 – 84 | 138 – 145 | 196 – 210 | 267 – 278 | 292 – 306 | 309 – 314 | 339 – 360 | 381 – 393 | 452 – 457 | 503 – 522 |
| **TuPV** | 73 – 85 | 139 – 146 | 197 – 213 | 197 – 213 | 295 – 309 | 312 – 317 | 342 – 363 | 384 – 397 | 456 – 461 | 507 – 526 |

**Amino acid residue number (based on VP2)**

**Supplementary Table 1.** Summary of variable regions (VRs) for RCPV and TuPV. Amino acid residue numbering based on the VP2 sequences.
